# School-Based Interventions for Promoting Physical Activity Using Games and Gamification: A Systematic Review Protocol

**DOI:** 10.3390/ijerph17145186

**Published:** 2020-07-17

**Authors:** Romina Gisele Saucedo-Araujo, Palma Chillón, Isaac J. Pérez-López, Yaira Barranco-Ruiz

**Affiliations:** 1 Department of Physical Education and Sports, PROFITH “PROmoting FITness and Health through Physical Activity” Research Group, Sport and Health University Research Institute (iMUDS), Faculty of Sport Sciences, University of Granada, 18071 Granada, Spain; pchillon@ugr.es; 2 Department of Physical Education and Sports, “Educación Física y Transformación Social”, SEJ546 Research Group, Faculty of Sport Sciences, University of Granada, 18071 Granada, Spain; isaacj@ugr.es; 3 Department of Physical Education and Sports, PROFITH “PROmoting FITness and Health through Physical Activity” Research Group, Sport and Health University Research Institute (iMUDS), Faculty of Education and Sport Sciences, University of Granada, 52071 Melilla, Spain; ybarranco@ugr.es

**Keywords:** exercise, AVG, children

## Abstract

Games and/or gamification seem to be a promising area for educational and health research. These strategies are being increasingly used for improving health indicators, even in educational settings; however, there is little information about these terms within the school to promote physical activity (PA). Objective: the aim of this study is to describe a systematic review protocol of school-based interventions for promoting PA in pre-schoolers, children, and adolescent students using games and gamification. Methods: This review protocol is registered in International prospective register of systematic reviews (PROSPERO) (CRD42019123521). Scientific databases include PubMed, Web of Science, SportDiscus, Cochrane Library, ERIC, and PsycINFO. A standardized procedure will be executed following the Preferred Reporting Items for Systematic Reviews and Meta-Analyses protocol (PRISMA-P) checklist for conducting systematic review protocols and the PICOS (Population, Interventions, Comparators, Outcomes, and Study design) tool to address an appropriate search strategy. Detailed information will be extracted, including a quantitative assessment using effect sizes to compare the interventions and a qualitative assessment using the Evaluation of Public Health Practice Projects (EPHPP) tool. Conclusion: This systematic review protocol contributes to establishing future systematic reviews using games and gamification strategies in school settings in order to examine their effect on PA outcomes among youth. Additionally, an update and clarification on the different terms in the school context have been included.

## 1. Introduction

In the last few decades, overweight and obesity have increased worldwide in adults [1,2] and in young people [3,4]. Furthermore, there has been a dramatic decrease in physical activity (PA) levels during childhood and middle adolescence. The World Health Organization recommends that children and adolescents practice at least 60 min a day of moderate to vigorous PA [5]. The health benefits of PA, specifically PA of moderate to vigorous intensity, have been frequently studied in school-age children and adolescents (5–17 years) [6,7]. Increased PA in children and adolescents has been positively associated with improvements in physiological and cognitive outcomes, such as body composition, cardiorespiratory fitness, bone health, cognition, and academic achievement [8]. Successful and attractive interventions are necessary to encourage increased levels of PA during childhood and adolescence. Furthermore, the school has proven to be an ideal setting for the development of these interventions to promote PA [9,10,11,12,13] for two main reasons: (a) the scholars’ age is key to changing habits, and young people with high levels of PA are more likely to be active adults [14]; and (b) schools provide compulsory academic training from childhood to adolescence that will ensure a universal education for every student.

### 1.1. Games and Gamification

A game, as Huizinga [15] suggested, is “a free, playful and essential activity for the human that is generally carried out for enjoyment or entertainment”. In the education field, when teachers design or adapt a game (especially a board game) for promoting learning in students, they use a methodology called game-based learning (GBL). An example would be “serious games or applied games”, which are designed primarily for learning and secondarily for entertainment [16]. Another term used in education is “exergame”, which is also known as an “active videogame” (AVG), referring to a game that requires PA in contrast to passive games [17] (e.g., conventional handheld games). Herein after, the term “AVG” will be used to refer to both AVGs and exergames to homogenize. In addition, AVGs rely on a technology that tracks body movement or reaction to the game in order to progress [18]. Previous studies have implemented serious games and AVGs in the educational context to increase PA levels from childhood to adolescence [19,20,21].

On the other side, a recent term related to games is gamification, which is often confused with GBL. Gamification is understood as the use of game elements in non-game contexts [22,23]. Gamification uses the dynamics and mechanics of a game to create a learning experience that increases students’ motivation. Thus, it increases their involvement and commitment to promote the desired behaviors. For this to happen, key aspects—such as missions, a narrative (around which the entire proposal is articulated), continuous feedback to the students, and decision-making processes by the students, among other elements—must be taken into account. Furthermore, gamification requires longer implementation times, because it is mainly oriented towards ambitious objectives, such as the development of certain competences, habits, or values in the students [24]. In contrast, GBL takes place only during specific periods of time because it usually focuses on a game designed with the primary objective of learning rather than entertaining [25]. Gamification has been a trending topic in different domains of knowledge such as health promotion [26,27,28], online programs [29], internet intervention [30], and education [31,32,33,34,35,36,37].

Therefore, the terms defined above are not synonymous but describe different valid approaches in the educational context that should not be confused because their focus and purpose are not the same [23,38]. Thus, games and gamification seem to be a promising area for education and health research [39,40], and they are starting to be used in educational contexts for different purposes. However, the school-based studies that implement games and/or gamification interventions can suffer from methodological weaknesses that generate problems in understanding how the interventions are implemented. In addition, there are many types of interventions; therefore, a systematic review is necessary to try to delve deeper into the subject, as well as the effects on the students.

### 1.2. Review Aim

Intervention studies in the scientific literature based on games and/or gamification are scarce, and there is little information on how to include this approach within educational contexts to achieve the expected goals. This systematic review protocol focuses on investigating school-based interventions using games and/or gamification to promote PA in young students. The first objective is to identify and examine school-based interventions using games and/or gamification to promote PA in pre-schoolers (3–5.99 years old), children (6–11.99 years old), and adolescents (12–18 years old) (non-university students). The second objective is to analyze their quality and effectiveness on the PA levels; accordingly, future recommendations will be provided to design and implement successful game and/or gamification interventions to promote PA in this population. Therefore, the first research question addressed in this systematic review is, “How many school-based interventions using games and/or gamification to promote PA are there, and how are they implemented?” The second question is, “What is the quality and effectiveness of these interventions, and what are the future recommendations?”

## 2. Methods

The protocol will be carried out strictly following the procedure of the PRISMA guidelines [41]. The systematic review protocol is in line with the items of the Preferred Networks for Systematic Review Initiatives and Meta-Analysis Protocols (PRISMA-P) (Supplementary Material Table S1). The PRISMA statement provides an evidence-based 27-item checklist for reporting systematic reviews and meta-analyses [42]. Following Cochrane’s guidelines on the development of a systematic review, the research questions formed the base of a search strategy that was further developed using PICOS (Population, Interventions, Comparators, Outcomes, and Study design) tool components (Table 1). The studies will be evaluated according to clearly defined criteria to determine their inclusion in or exclusion from the review, and the findings from included studies will be evaluated and reported [43].

### 2.1. Eligibility

The inclusion criteria for the studies will be (a) to report PA as a primary or secondary outcome; (b) to implement a game and/or gamification intervention within the school; (c) intervention studies where a game and/or gamification were included with the learning objective; (d) to target pre-schoolers, children, and/or adolescent students (non-university students); and (e) to be written in English or Spanish. In the latter case, the abstract must also be written in English.

### 2.2. Outcomes

The outcome will be PA, which may be measured objectively (such as using an accelerometer or pedometer) or self-reported and/or through observation. Examples of potential PA outcomes would be minutes of moderate to vigorous PA, minutes or counts of total PA, total steps, or any other qualitative values (such as Likert scale).

### 2.3. Information Sources

For this purpose, the included databases will be PubMed, Web of Science, SPORTDiscus, Cochrane Library, ERIC, and PsycINFO, because there are previous review studies on this topic that have used some of these electronic databases [26,44,45]. In addition, one of the authors (I.J.P.-L.) is an expert and investigates the games and/or gamification field and will provide appropriate insights and potential studies to include. In addition, a manual search will be performed to identify studies that were not selected in the electronic research, and the references of these articles will be verified for the same purpose.

### 2.4. Search Strategy

The search will be conducted following the PRISMA guidelines [42]. The electronic search will be conducted in July 2020. Five categories of search terms will be identified: (a) games (serious games and applied games and AVGs) and gamification, (b) physical activity, (c) school, (d) intervention, and (e) age. The terms for each category will be obtained by consulting other reviews, such as (a) serious games and AVG [19,20,21,46,47,48,49,50], gamification [26,40,51,52], (b) physical activity [53,54], and (c) intervention [55,56]. Regarding the high diversity of game-related terms used in the literature, the most used and standardized terms found in the scientific references will be included in the current review. For example, the electronic search will be conducted following these sequences of terms: “game* OR ‘game based learning’ OR ‘GBL’ OR video gam* OR mobile gam* OR exergam* OR ‘AVG’ OR active video gam* OR gamifi* OR gamification OR ‘serious games’ OR ‘applied games’ OR app gam* AND ‘physical activity’ OR walk OR steps OR ‘physical fitness’ OR ‘leisure activity’ OR ‘motor activity’ OR exercise OR training OR sport AND school OR kindergar* OR ‘high school’ OR ‘nursery school’ AND intervention* OR program* OR school-based AND preschool* OR child* OR adolescen* OR young* OR youth OR kid OR teenage” (Supplementary Material Table S2). The search strategy will be carried out within each database, including PubMed [Title/Abstract], Web of Science [Topic], SportDiscus [Title], Cochrane Library [Title Abstract Keyword], ERIC [abstract], and PsycINFO [abstract].

### 2.5. Selection Process

After performing the search in the electronic databases, all the identified records will be uploaded through EndNote X7 -a reference management software- (Thomson Reuters, New York, NY, USA). Then, the authors will easily identify any duplicates and will delete them. After duplicates are removed, all titles and abstracts will be evaluated for eligibility by two reviewers (R.G.S.-A. and Y.B.-R.) using the established inclusion criteria, and for those that are not clear, full texts will be searched. After obtaining the first full texts of potentially eligible studies, two reviewers (R.G.S.-A. and Y.B.-R.) will independently review the full texts based on the eligibility criteria. They will perform the screening and agree by consensus, and any disagreement in the inclusion process will be resolved with the rest of the authors.

### 2.6. Extraction and Synthesis of the Data

Two reviewers (R.G.S.-A. and Y.B.-R.) will verify the data extraction to check its completeness and accuracy. The results will be organized in chronological order, and tables will be created to capture the information. The following data will be extracted: descriptive information such as the authors and country, sample size and age (years), intervention study design and duration, the games or gamification used, PA outcomes, results from PA outcomes, and results from other outcomes.

### 2.7. Quality Assessment

Two researchers will conduct a quality assessment of the identified studies. The quality assessment will be carried out using a standardized evaluation framework called “Effective Public Health Practice Project” (EPHPP) [52,57] (http://www.ephpp.ca/tools.html). The EPHPP is a generic tool to evaluate a variety of study designs of intervention studies, studies such as RCT, before and after studies, and case-control studies. This tool is suitable for use in systematic reviews to check the effectiveness of interventions [54] and is used especially for health promotion and public health interventions [52]. This tool evaluates six methodological dimensions: selection bias, study design, confounding factors, blinding, data collection methods, withdrawals, and dropouts, all of which include a global rating. The EPHPP quality assessment tool includes a standardized dictionary developed to classify factors as weak, moderate, or strong. The individual grades of each study are used to calculate a global score that assigns a total grade: (a) weak (when two or more factors are rated as weak); (b) moderate (when one factor is rated as weak and four factors are rated as strong); and (c) strong (when there are no weak ratings). Two researchers (R.G.S.-A. and Y.B.-R.) will read and evaluate the quality of the articles according to the “Quality articles” criteria. Disagreements will be resolved by discussing the different opinions with the rest of the authors until a common opinion is reached.

### 2.8. Risk of Bias

Following Gunnell et al. [58], we will assess the risk of bias through ‘The Cochrane Risk of Bias Tool 2’ for randomized studies and ‘ROBINS-1’ for non-randomized studies. The Cochrane Risk of Bias Tool 2 [59] includes five items: (1) bias arising from the randomization process; (2) bias due to deviations from intended interventions; (3) bias due to missing data; (4) bias in measurement outcomes; and (5) bias in the selection of the reported result. In addition, the rating system for each domain is either ‘low’, ‘high’, or ‘unclear’ risk of bias, and the researcher makes a judgment with supporting statements about the risk of bias [59]. The ROBINS-I tool domains [60] include bias due to (1) confounding, (2) selection of participants for the study, (3) classification of interventions, (4) deviations from intended interventions, (5) missing data, (6) measurement of outcomes, and (7) selection of the reported results, and risk of bias is assessed as ‘low’, ‘moderate’, ‘serious’, or ‘critical’.

### 2.9. Effectiveness

In regards to data analysis and synthesis, if the data extracted are available, the researchers will conduct a meta-analysis to determine the effect of school-based interventions for promoting PA using games and gamification in comparison to the control groups (i.e., no intervention). For studies that include different types of interventions for comparison, not including control group, data will be also examined separately to the previous comparison analysis. The effect size of each study will be calculated as standardized mean difference (post minus pre) expressed as Hedges’ g to correct for possible small sample bias [61]. Finally, the effect size of all studies included will be combined to estimate an overall effect with a 95% confidence interval. Fixed- or random-effects models will be selected based on the heterogeneity of the studies examined. The heterogeneity will be evaluated using the I^2^ statistics. Additionally, we will examine the one-leave-out analysis in order to check the robustness of the estimates. Finally, to detect publication bias, we will conduct a visual inspection of funnel plots and the Egger test [62]. All the analysis will be performed using STATA 13 (StataCorp. 2013. Stata Statistical Software: Release 13. College Station, TX: StataCorp LP) software.

## 3. Data Reporting

In accordance with the PRISMA-P recommendations, the protocol for this systematic review was developed and submitted by the authors for registration in PROSPERO (registration number: CRD42019123521) and is available online (http://cort.as/-GZHe). The systematic review will follow Cochrane’s recommendations and will be reported based on the PRISMA guidelines.

## 4. Expected Results

This protocol intends to present the specific methodology that will be used in a systematic review to identify and examine school-based intervention studies focused on game and/or gamification strategies to promote PA. The included studies will provide preliminary evidence regarding games or gamification as novel and attractive strategies to improve PA levels in the youth population in a school setting.

PA patterns are developed from childhood to adulthood [63,64]. Along these stages, most children and adolescents spend the majority of the day at school. Thus, the school becomes an ideal and appropriate environment to promote PA in children and adolescents [65]. Moreover, school has an influence on children during the transition from childhood to youth [66]. In recent years, evidence suggests that PA interventions at school can be effective for increasing PA in children and adolescents [37,67,68,69,70]. In addition, there is evidence that PA in the classroom may have a positive impact on academic results [55], levels of concentration, memory, and classroom behaviors [71].

A preliminary search found several studies based on game and/or gamification strategies that reported PA outcomes in a school context. For example, AVGs appear to hold promise as a method for increasing PA. This can improve health status and offer other social and academic benefits [72]. Accordingly, the systematic review by Williams et al. [46] concluded that AVGs can be an effective means of increasing adolescents’ overall PA levels. Moreover, a 12-week school-based intervention (60 min/twice per week) based on AVGs improved Chinese children’s PA levels [73]. Likewise, students from a public elementary school who participated in an AVG intervention reported higher PA compared with those students who maintained their regular physical education lessons [74].

The use of the term gamification has been increasing, and it has been implemented for different age groups. In particular, one example of gamification is the study by Coombes et al. [35], which implemented “Beat the Street”, a 9-week pilot intervention study in children from 8 to 10 years old. They evaluated the impact of touching smart cards with sensors to promote active travel to school and thus increase PA through competition, scores, and awards. The intervention did not significantly impact children’s overall PA levels during school commute times, but there was evidence that the intervention had a positive impact on higher intensity PA during the commute to school. Another study carried out three types of interventions in a period of 20 weeks [33], and one of the interventions was gamified. During the gamified weeks, students received challenging weekly objectives; when each class met its objective, a new objective was implemented. Therefore, daily, weekly, and post-intervention incentives were offered to reinforce positive behavior among students and even the possibility of earning badges, trophies, a personalized shirt, and a 2-h field day after the intervention. This study concluded that gamification is an important and promising strategy to increase PA in the classroom. A secondary school in Northern Ireland carried out a gamified intervention [75]. The StepSmart Challenge was a 24-week intervention in adolescents to change behavior related to PA. The design of this intervention was based on team and individual competition using motivation and incentives. In addition, in the United Kingdom [76], a pilot evaluation of a community-wide gamification-based PA intervention was applied to the city/town and also called “Beat the Street”. Participants recorded their walking and cycling tours by tapping “Beat Boxes” to record their PA levels. The participants received points and several incentives. This pilot study provides preliminary evidence that the intervention could increase PA levels.

Gamification strategies have also been implemented with university students. A gamification program using a mobile app at a university reported significant effects on cardiorespiratory fitness compared to the control group that followed a traditional teaching methodology [31]. Accordingly, another study about a gamification-based teaching program designed to increase PA levels in college students showed a significant effect on their cardiorespiratory fitness in comparison with peers of the control group [32].

Despite the recent studies based on games and gamification, there is no full review following a rigorous process to identify the overall school-based interventions focused on these strategies. In addition, research is needed to assess the impact of games and/or gamification strategies on the PA levels of young people. Regarding the importance of maintaining the effect of the intervention at the school level once the researchers are not present, as suggested by Sallis et al. [77], it seems that gamification provides positive effects. However the effects are highly dependent on the context in which gamification is implemented, as well as on the study participants and the effectiveness of the interventions [28,40]. In addition, a recent systematic review protocol focused on the term ‘gamification’ has been published [45]. However, because there is great terminological and methodological confusion in the use of these strategies based on games to increase PA levels, it seems more appropriate to include all the terms mentioned in the current study.

With this research, it is expected that more future school-based interventions using games or gamification may be implemented using appropriate methodologies to be effective in the classroom. Therefore, this review will help clarify the relationship between game and/or gamification strategies with PA improvements. Important guidelines will be provided for future research in the area of PA in order to optimize the educational value of games and/or gamification. Games and/or gamification could be a promising strategy representing an excellent alternative, although regarding gamification, there is still a dearth of valid empirical evidence in this field.

## 5. Limitations and Strengths

The limitations of the review protocol are the language inclusion criteria including only English and Spanish. Despite RCTs would be the ideal studies design to include in systematic reviews, is important taking in account that in the present systematic review protocol all types of studies design will be included (even studies with no controls) due to it is a novel field of research and in an educational context, where the application of studies with RCTs design are scarce. Regarding the strengths, to our knowledge, there is no review that fills these gaps in the literature by performing a broad search to find interventions based on game and gamification strategies.

## 6. Conclusions

A detailed description of the systematic review protocol on school-based interventions to promote PA in pre-schoolers, children, and adolescent students using games and/or gamification has been presented in this manuscript. The review methodology—including recommended electronic database searching, identification of records, screening, and data extraction strategies, as well as recommended tools for quality and effective analyses of intervention studies—has been described in detail. Additionally, an update and clarification on the different terms related to games and gamification in the school context has been included.

## Figures and Tables

**Table 1:** PICOS tool components.

Population	Students from 3 to 18 years old.
Interventions	School-based.
	Games and/or gamification to promote physical activity or exercise.
Comparators	Pre-to-post, pre- and post- treatment comparisons ± with/without controlled group.
Outcomes	Physical activity.
Study design	Experimental studies such as randomized clinical trials (RCTs), controlled clinical trial (CCT), quasi-experimental non-randomized studies, and pre-post intervention studies without control group.

PICOS—Population, Interventions, Comparators, Outcomes, and Study design.

## Supplementary Materials

The following are available online at https://www.mdpi.com/1660-4601/17/14/5186/s1, Table S1: PRISMA-P (Preferred Reporting Items for Systematic Review and Meta-Analysis Protocols) 2015 checklist: recommended items to address in a systematics review protocol; Table S2: Search strategy.
